# Comparative Analysis of Metabolic Differences of Jersey Cattle in Different High-Altitude Areas

**DOI:** 10.3389/fvets.2021.713913

**Published:** 2021-08-03

**Authors:** Zhiwei Kong, Bin Li, Chuanshe Zhou, Qinghua He, Yuzhong Zheng, Zhiliang Tan

**Affiliations:** ^1^Department of Food Science and Engineering, College of Chemistry and Environmental Engineering, Shenzhen University, Shenzhen, China; ^2^Key Laboratory of Optoelectronic Devices and Systems of Ministry of Education and Guangdong Province, College of Optoelectronic Engineering, Shenzhen University, Shenzhen, China; ^3^School of Food Engineering and Biotechnology, Hanshan Nornal University, Chaozhou, China; ^4^Institute of Animal Husbandry and Veterinary, Tibet Autonomous Regional Academy of Agricultural Sciences, State Key Laboratory of Hulless Barley and Yak Germplasm Resources and Genetic Improvement, Lhasa, China; ^5^CAS Key Laboratory for Agro-Ecological Processes in Subtropical Region, National Engineering Laboratory for Pollution Control and Waste Utilization in Livestock and Poultry Production, Hunan Provincial Key Laboratory of Animal Nutritional Physiology and Metabolic Process, Institute of Subtropical Agriculture, The Chinese Academy of Sciences, Changsha, China

**Keywords:** metabonomics, high altitude, Jersey cattle, difference, hypoxia

## Abstract

In high-altitude area, hypoxia is a serious stress for humans and other animals, disrupting oxygen homeostasis and thus affecting tissue metabolism. Up to now, there are few reports on the metabolic changes of dairy cows at different altitudes. In this experiment, metabonomics technology and blood biochemical indexes were used to study the metabolic changes of dairy cows in different altitudes. The results showed that the different metabolites were mainly enriched in amino acid metabolism and sphingolipid metabolism, and sphingolipid metabolism showed a negative correlation with increased altitude. The results of this study will enrich the hypoxia-adaptive mechanism of dairy cows in high-altitude areas and provide a theoretical basis for the nutritional regulation of performance and disease treatment of dairy cows in high-altitude areas.

## Introduction

From the medical point of view, there is no obvious geological boundary between high and low altitudes, but from the evidence of plateau-related diseases, it is generally believed that high altitude (HA) refers to the altitude of 1,500 m or above the average sea level (1). It can be divided into three levels: high altitude (1,500–3,500 m), extremely high altitude (3,500–5,500 m), and extreme altitude (>5,500 m) (2). The main challenge faced by vertebrates at high altitude is that the reduction of oxygen partial pressure (PO_2_) limits the aerobic metabolic rate, which leads to metabolic adaptation to reduce oxygen demand (3). At present, the research on animal metabolism adaptability at high altitude mainly includes rats (4), pigs (5), donkeys, and sheep (6). There are few reports on the metabolic adaptation of dairy cows in high-altitude areas, and the description of metabolic differences of dairy cows between different altitudes is less.

As an important breed of dairy cattle in countries with developed animal husbandry, Jersey cattle has the characteristics of rough feeding tolerance, strong disease resistance, and strong adaptability (7). In addition, Jersey cattle's milk fat color is yellow, its fat globules are large and easy to separate, and it is an ideal raw material for processing high-quality cream (8). Butter (mainly composed of milk fat) provides rich nutrients for residents in high-altitude areas and is an indispensable life product. What is more, it was found that the adaptability of Jersey cattle was the best among the different breeds introduced to high-altitude areas. Therefore, there is a lot of interest in Jersey cattle adapting to high altitudes.

Recent studies have found that people from low altitude to high altitude will cause significant remodeling of tissue metabolism, as well as changes in the level of circulating metabolism (9, 10). Hypoxia can inhibit the oxidative metabolism of heart (11) and skeletal muscle (12), reduce the ability of fatty acid oxidation (13), and increase glycolysis (14) in rodents and non-plateau native people. Based on miRNA and proteomics, we found that Jersey cattle adapt to high-altitude hypoxia by regulating inflammatory homeostasis (15). However, the specific metabolic adaptation mechanism of Jersey cattle transferred from low altitude to high altitude is still unclear. Metabonomics based on mass spectrometry (MS) is a method to study the overall changes of small-molecule metabolites, reflecting the physiological activities in organisms (16). In this study, metabonomics was applied to explore the metabolic changes of Jersey cattle in different altitudes. This will enrich the adaptive mechanism of Jersey cattle at high altitude and lay a theoretical basis for subsequent nutritional regulation.

## Materials and Methods

This study was carried out based on the animal protection and use guidelines of the Animal Protection Committee, Institute of Subtropical Agriculture, Chinese Academy of Sciences (protocol ISA-201809).

### Animals and Experimental Design

Eighteen multiparous Jersey cattle (400 ± 28 kg) were selected and randomly divided into three groups (six cattle in each group) in Shenyang [altitude 50 m; high-altitude-free (GJ) group], Nyingchi [altitude 3,000 m; high-altitude (CJ) group], and Lhasa [altitude 3,650 m; extremely high-altitude (XJ) group] for 60 days in the autumn. Six animals were randomly selected from each group for metabonomics analysis and other measures. Based on the Chinese Feeding Standard of Dairy Cow (MOA, 2004), basic diets meeting the nutritional requirements of energy, protein, minerals, and vitamins are prepared (Table 1). The same TMR diet was fed *ad libitum*.

**Table 1:** The difference of plateau adaptability of dairy cows at different altitudes.

**Items**	**Treatment**			**SEM**	***P*-value**
	**GJ**	**CJ**	**XJ**		
BOS (%)	90.08	84.55	72.48	1.541	0.001
NO (μmol/l)	168.03	202.62	184.45	6.571	0.006
NOS (U/ml)	1,607.45	1,863.94	1,744.45	44.636	0.004
EPO (mU/ml)	4,838.19	5,254.33	5,689.11	144.903	0.003
HSP70 (ng/ml)	19.34	19.81	20.13	0.233	0.039
HIF-1 (ng/l)	138.33	165.44	193.66	13.501	0.018
VEGF (ng/ml)	167.05	185.44	207.32	7.659	0.006

*BOS, blood oxygen saturation; NO, nitric oxide; NOS, nitric oxide synthase; TRP, total reactive protein; EPO, erythropoietin; HSP70, heat shock protein 70; VEGF, vascular endothelial growth factor; HIF, hypoxia-inducible factor; GJ, high-altitude-free group; CJ, Nyingchi (altitude 3,000 m; high-altitude group); XJ, Lhasa (altitude 3,650 m; extremely-high-altitude group)*.

### Measurement of Blood Oxygen Saturation

After the vulva of the cow was opened, and the liquid was dried with an absorbent paper. The blood oxygen saturation was measured with Nonin Avant 9600 (Nonin Medical, Inc., Plymouth, MN, USA) blood oxygen saturation detector. The sensor probe was close to the vulva skin to measure the blood oxygen saturation (BOS). The average of the three measurements is taken as the measurement value.

### Blood Sample Preparation

Before the morning feeding on the last day of the experiment, all cows were punctured through the caudal vein to take blood samples. The blood samples in the anticoagulant tube were centrifuged at 3,000 rpm for 10 min at 4°C. The plasma was collected and stored in a refrigerator at −80°C for metabonomics analysis. The blood samples collected by a non-anticoagulant tube were centrifuged at 2,500 rpm for 5 min. The serum was collected and stored in a refrigerator at 4°C for determination of biochemical indexes.

### High-Altitude Adaptation Index Determination

The levels of nitric oxide (NO), nitric oxide synthase (NOS), total reactive protein (TRP), erythropoietin (EPO), heat shock protein 70 (HSP70), vascular endothelial growth factor (VEGF), and hypoxia-inducible factor (HIF) in serum were measured by ELISA kit.

### Metabolite Extraction

Firstly, the 100 μl plasma obtained by centrifugation was mixed with 300 μl methanol (including internal standard 1 μg/ml), vortexed for 30 s, sonicated in ice bath for 10 min, and incubated at −20°C for 1 h to precipitate protein. Secondly, to process the sample, it was centrifuged at 12,000 rpm for 15 min at 4°C. Finally, the supernatant was transferred to a liquid chromatography–mass spectrometry (LC/MS) sample bottle at −80°C for storage and standby and used for the analysis of UHPLC-QE Orbitrap/MS. Quality control (QC) samples were prepared by mixing the same supernatant from all samples.

### Liquid Chromatography With Tandem Mass Spectrometry Analysis

A liquid chromatography with tandem mass spectrometry (LC–MS/MS) analysis was performed using a UHPLC system (1290, Agilent Technologies, Santa Clara, CA, USA) coupled with a UPLC HSS T3 column (2.1 mm × 100 mm, 1.8 μm) with Q Exactive (Orbitrap MS, Thermo Fisher Scientific, Waltham, MA, USA). Mobile phase A was positive in 0.1% formic acid aqueous solution and negative in 5 mmol/l ammonium acetate aqueous solution, and mobile phase B was acetonitrile. The elution gradient was set as following: 0 min, 1% B; 1 min, 1% B; 8 min, 99% B; 10 min, 99% B; 10.1 min, 1% B; and 12 min, 1% B. The flow rate was 0.5 ml/min. The injection volume is 3 μl. In LC/MS experiments, QE mass spectrometer can obtain MS/MS spectra on an information-dependent basis (IDA). In this mode, the acquisition software (Xcalibur 4.0.27, Thermo Fisher Scientific, Waltham, MA, USA) continuously evaluates full-scan measured MS data while collecting and triggering MS/MS spectral acquisition according to pre-selected criteria. ESI source conditions were set as follows: sheath gas flow rate of 45 arb, auxiliary gas flow rate of 15 arb, capillary temperature of 400°C, full MS resolution of 70,000, MS/MS resolution of 17,500, impact energy of 20/40/60 eV, and injection voltage of 4.0 kV (positive) or −3.6 kV (negative).

### Statistical Analysis

ProteoWizard was used to convert the original data into mzXML format and processed by MAPS software (version 1.0). A data matrix consisting of retention time (RT), mass/charge ratio (*M*/*Z*), and peak strength was generated. The internal MS2 database was used for metabolite identification. The card value standard of differential metabolites was that a *p*-value of Student's *t*-test was < 0.05. Meanwhile, the variable importance in the projection (VIP) of the first principal component of OPLS-DA model is >1.

## Results

### Physiological and Biochemical Characteristics

The difference of plateau adaptability of dairy cows at different altitudes is shown in Table 1. Compared with cows at low altitude (GJ), blood oxygen saturation of cows at high altitude (CJ) and extremely high altitude (XJ) was significantly lower (*P* < 0.05), and blood oxygen saturation of cows at extremely high altitude was significantly lower (*P* < 0.05) than that of cows at high altitude. In addition, the level of NO, NOS, EPO, HSP70, HIF-1, and VEGF decreased (*P* < 0.05) significantly with the increase of altitude.

### Overview of Differential Metabolomic Profiles

**Figure 1:**
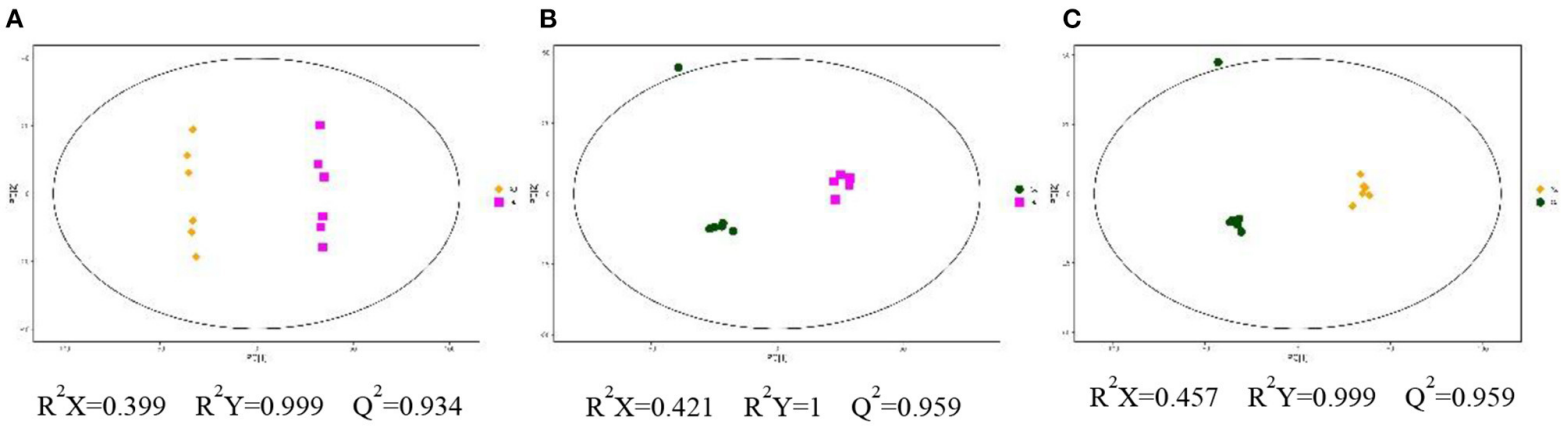
PLS-DA score plot of the three groups in plasma of Jersey cattle using the identified metabolites in positive ionization mode. **(A)** The difference of CJ vs. GJ; **(B)** the difference of XJ vs. GJ; **(C)** the difference of CJ vs. XJ.

**Figure 2:**
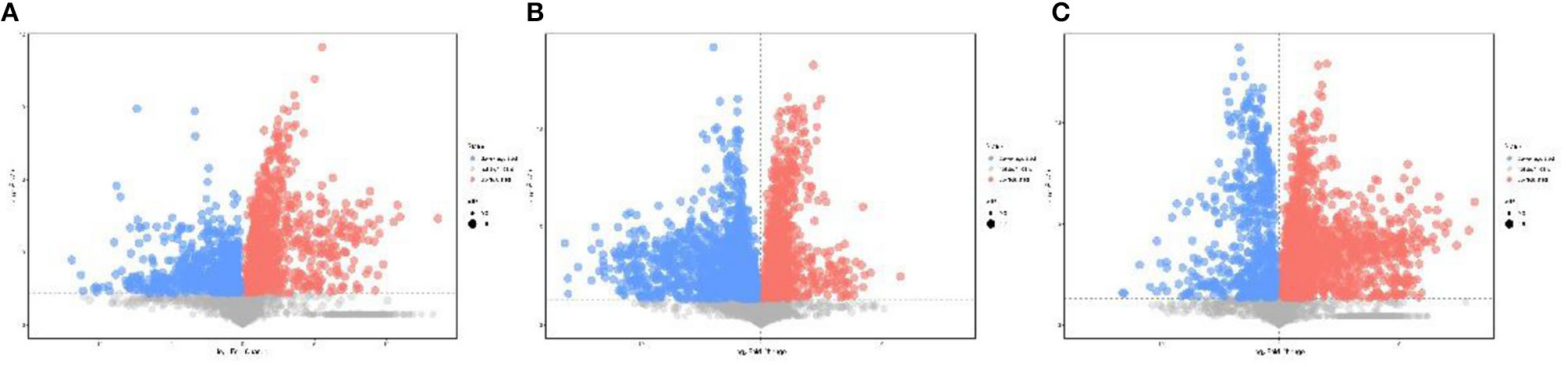
Volcanic plots of the three groups in plasma of Jersey cattle using the identified metabolites in positive ionization mode. **(A)** The results of CJ vs. GJ; **(B)** the results of XJ vs. GJ; **(C)** the results of CJ vs. XJ.

In positive ionization mode, compared with the GJ group, a total of 105 and 103 differential metabolites (VIP > 1, *p* < 0.05) were found in the CJ and XJ groups (Supplementary Table 1). In addition, 124 differential metabolites were identified in the CJ group compared with the XJ group (Supplementary Table 1). Partial least-squares discriminant analysis (PLS-DA) was performed to obtain a global overview of the differences in metabolites among the three groups (Figure 1). The *R*^2^*Y* and *Q*^2^ values of the PLS-DA models are all above 0.93. The above results indicate that exposure to various altitudes can interfere with the metabolism of dairy cows, which is also supported by the observation results of volcanic plots (Figure 2).

### Comparison of Metabolomic Profiles Among the Three Altitudes

**Figure 3:**
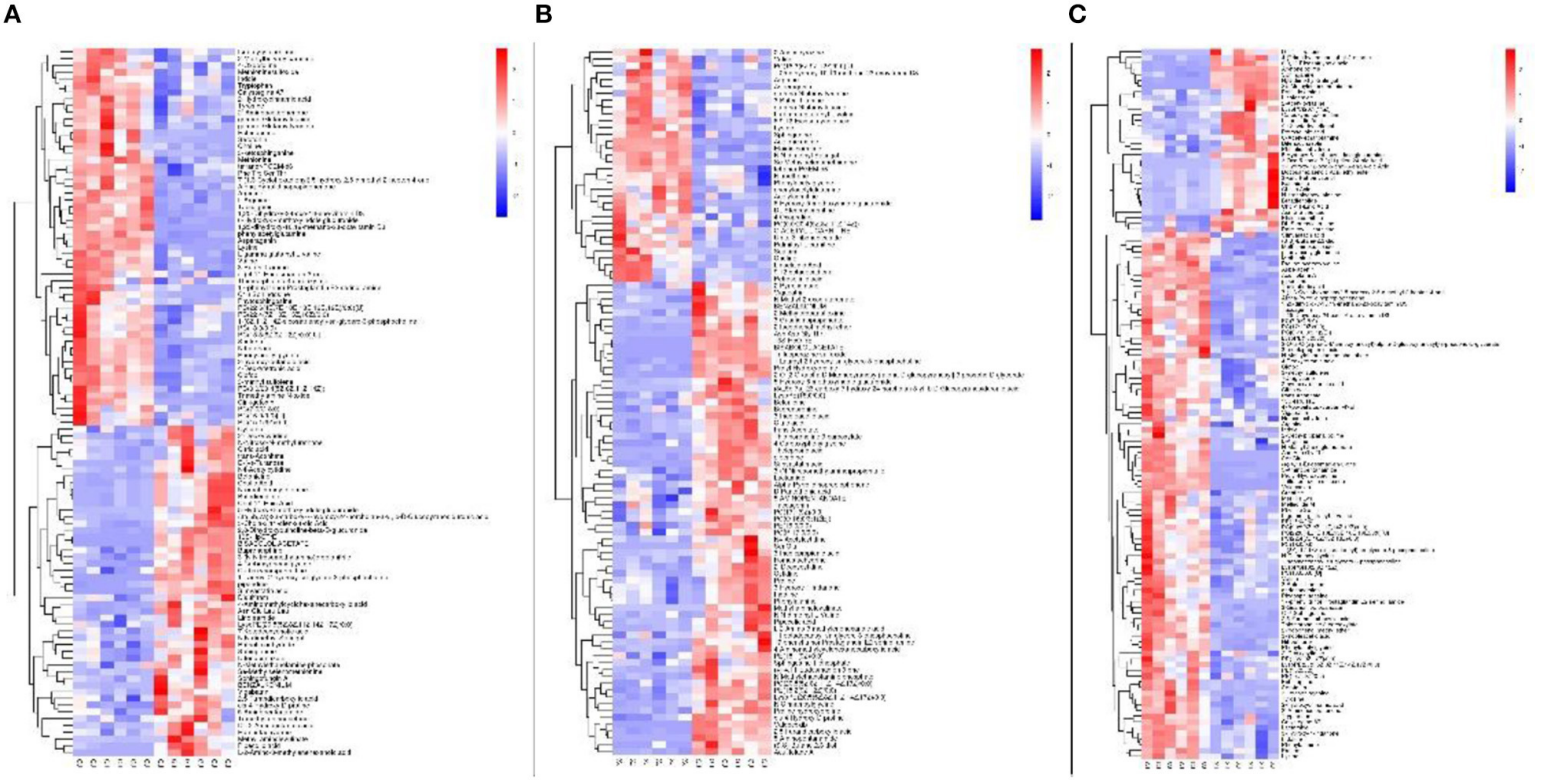
Heatmap of hierarchical clustering analysis for the three groups in palsma of Jersey cattle using the identified metabolites in positive ionization mode. **(A)** The clustering results of CJ vs. GJ; **(B)** the clustering results of XJ vs. GJ; **(C)** the clustering results of CJ vs. XJ.

**Figure 4:**
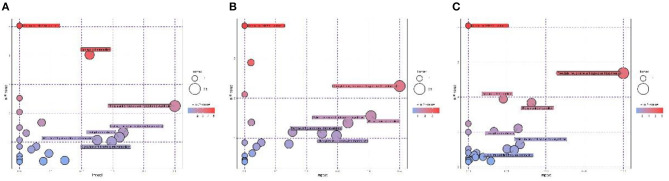
Pathway analysis of the identified metabolites in positive ionization mode in plasma of Jersey cattle exposed to different altitudes. **(A)** The pathway analysis results of CJ vs. GJ; **(B)** the pathway analysis results of XJ vs. GJ; **(C)** the pathway analysis results of CJ vs. XJ.

In positive ionization mode, the heat map constructed from the 105, 103, and 124 differential metabolites revealed two diverse metabolomic profiles of various altitudes (Figure 3). Kyoto Encyclopedia of Genes and Genomes (KEGG) enrichment analysis indicated that up-regulated metabolites in the CJ group were mainly enriched in aminoacyl-tRNA biosynthesis; sphingolipid metabolism; phenylalanine, tyrosine, and tryptophan biosynthesis; and valine, leucine, and isoleucine biosynthesis (Figure 4A). Up-regulated metabolites in the XJ group were enriched in aminoacyl-tRNA biosynthesis; phenylalanine, tyrosine, and tryptophan biosynthesis; phenylalanine metabolism; and valine, leucine, and isoleucine biosynthesis (Figure 4B). In addition, compared with the XJ group, the up-regulated pathways were mainly aminoacyl-tRNA biosynthesis; phenylalanine, tyrosine, and tryptophan biosynthesis; sphingolipid metabolism; and phenylalanine metabolism in the CJ group (Figure 4C). The details of all enriched pathways are shown in Supplementary Table 2.

### Key Metabolite Identification

**Figure 5:**
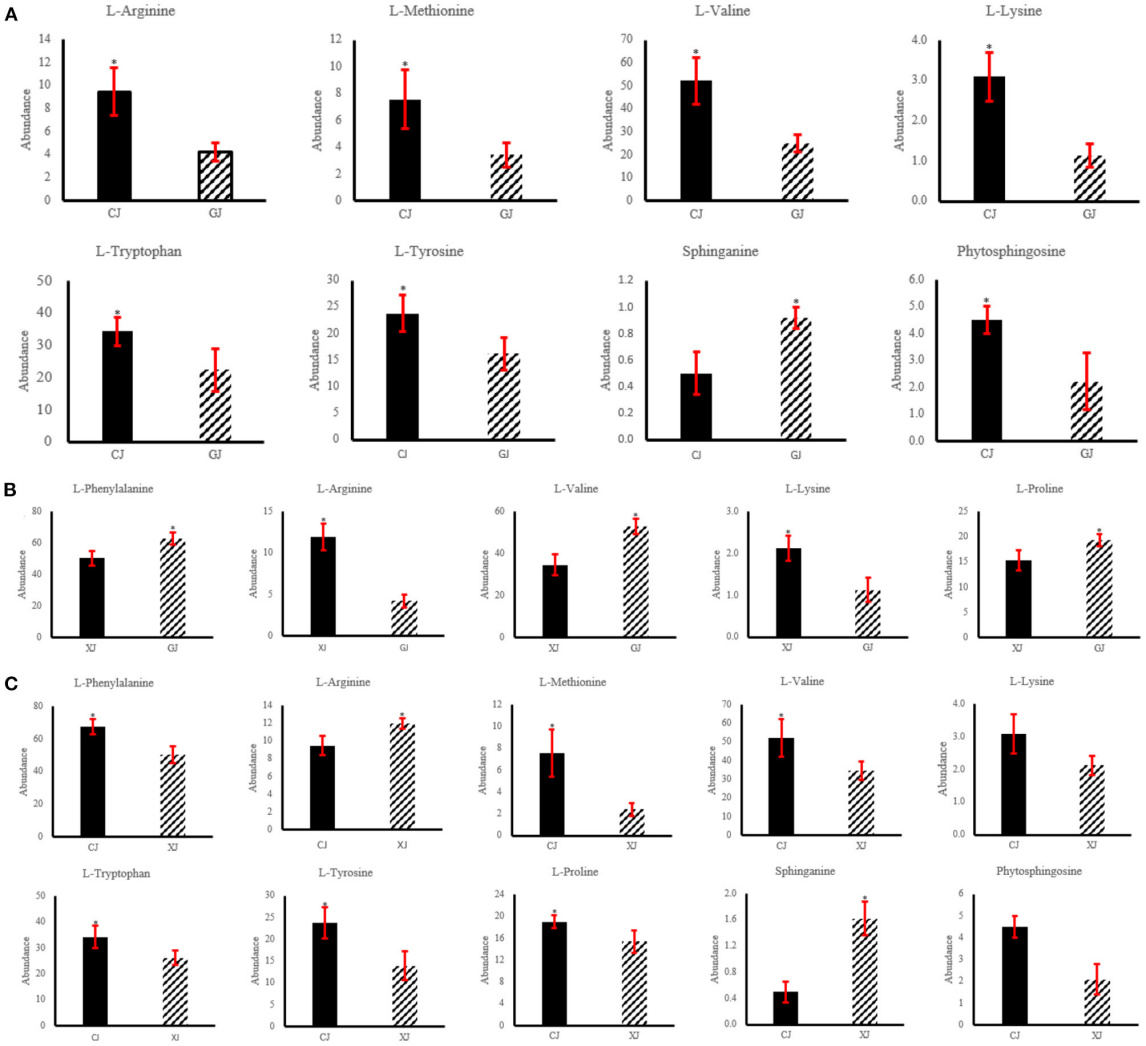
Differential metabolite abundances in plasma of Jersey cattles at different altitudes. **(A)** The abundance of identified differential metabolites for CJ VS GJ in positive ionization mode. “CJ and GJ” represent high altitude and control group, respectively; **(B)** the abundance of identified differential metabolites for XJ VS GJ in positive ionization mode. “xJ and GJ” represent extremely high altitude and control group, respectively; **(C)** the abundance of identified differential metabolites for CJ VS XJ in positive ionization mode. *n* = 6, *p < 0.05.

Metabolites that could be utilized to distinguish the CJ group from the XJ group were identified on the basis of VIP > 1, FC > 1, and *p* < 0.05. L-methionine, L-tryptophan, L-arginine, and L-lysine in the aminoacyl-tRNA biosynthesis pathway; L-tyrosine in phenylalanine; tyrosine and tryptophan biosynthesis and L-valine in valine; leucine; and isoleucine biosynthesis showed higher abundance (*P* < 0.05) in the CJ group than in the GJ and XJ groups (Figure 5 and Table 2). Phytosphingosine in sphingolipid metabolism showed higher abundance (*P* < 0.05) in the CJ group than in the GJ and XJ groups (Figures 5A,C and Table 2), while sphinganine in sphingolipid metabolism and L-proline in aminoacyl-tRNA biosynthesis and L-phenylalanine in phenylalanine, tyrosine, and tryptophan biosynthesis showed lower abundance (*P* < 0.05) in the XJ group than in the GJ group (Figure 5B and Table 2). In addition, L-proline in aminoacyl-tRNA biosynthesis and L-phenylalanine in phenylalanine, tyrosine, and tryptophan biosynthesis showed higher abundance (*P* < 0.05) in the CJ group than in the XJ group (Figure 5C and Table 2).

**Table 2:** Differentially expressed metabolites of different groups.

**Group**	**MS2 name**	**VIP**	***P*-value**	**Fold change**
CJ vs. GJ	L-Methionine	1.6063	0.0003	2.1971
	L-Arginine	1.8007	0.0000	2.8363
	L-Valine	1.6853	0.0001	2.0993
	L-Lysine	1.7777	0.0000	2.7309
	L-Tryptophan	1.5732	0.0006	1.5375
	L-Tyrosine	1.3827	0.0056	1.4700
	Phytosphingosine	1.3112	0.0424	2.0365
	Sphinganine	1.3867	0.0119	0.5381
XJ vs. GJ	L-Arginine	1.5491	0.0001	2.2398
	L-Valine	1.1395	0.0213	1.3945
	L-Lysine	1.4544	0.0003	1.8684
	L-Proline	1.0817	0.0198	0.7967
	L-Phenylalanine	1.1146	0.0189	0.8009
CJ vs. XJ	L-Phenylalanine	1.3532	0.0008	1.3407
	L-Arginine	1.0112	0.0019	1.9071
	L-Methionine	1.5442	0.0003	3.1485
	L-Valine	1.3466	0.0013	1.5054
	L-Lysine	1.3119	0.0012	1.4616
	L-Tryptophan	1.2364	0.0072	1.3042
	L-Tyrosine	1.5141	0.0003	1.7095
	L-Proline	1.1753	0.0080	1.2348
	Phytosphingosine	1.2049	0.0359	2.1468
	Sphinganine	1.5114	0.0000	0.3056

*GJ, high-altitude-free group; CJ, Nyingchi (altitude 3,000 m; high-altitude group); XJ, Lhasa (altitude 3,650 m; extremely-high-altitude group)*.

## Discussion

The oxygen partial pressure in the atmosphere is 60% that of the sea level. The dairy cows imported into the area under low oxygen environment have a higher incidence rate of altitude sickness. Since the beginning of the last century, the introduction of Holstein dairy cows from the mainland in Tibet has failed. Later, due to the strong adaptability of Jersey cattle, we introduced Jersey cattle and achieved good results. Our previous research explored its adaptive mechanism from the miRNA and proteome level (15). This paper further elaborated its good adaptive mechanism from the metabolic level, as well as the adaptive differences at different altitudes.

The results showed that the blood oxygen saturation of Jersey cattle in high-altitude and very-high-altitude areas decreased with the increase of altitude, which may be due to the functional damage of lung tissue caused by high altitude, resulting in the decrease of lung oxygen supply and the obstruction of carbon dioxide exhalation, which eventually led to more severe hypoxia in dairy cows with the increase of altitude (17). Hypoxia can promote the production of vasodilator *in vivo*, and vasodilator can inhibit the formation of vascular injury and pulmonary hypertension caused by hypoxia (18). NO is a very effective vasodilator (19), which can promote vasodilation, increase blood flow velocity, and prevent vascular remodeling caused by hypoxia (20). Studies have shown that hypoxia can increase the expression of NOS, which can be used to explain the findings of this experiment that the level of NOS increases with altitude (21). In addition, the level of NO also increased with the elevation, which may be due to the increase of the expression of nitric oxide, which is the core mechanism of mammalian adaptation to hypoxia (22, 23). However, the level of NO and NOS was higher in the CJ group than in the XJ group, which might resulted from downregulation of arginine and proline metabolism leading to the production of oxidative stress (24). The results showed that the expression of HIF was up-regulated under hypoxia (25), which was consistent with the results of this experiment. HIF can increase the expression of EPO after it enters the cells (26), so it can adapt to high-altitude hypoxia by increasing the oxygen-carrying capacity of the body (27). Therefore, the higher the altitude is, the higher the level of EPO is, as was shown in our results. VEGF is recognized as the most typical target gene of HIF-1 α (28). In this study, it was found that HIF-1 was up-regulated with the increase of altitude, which may be because HIF-1 regulates VEGF to promote the formation of blood vessels to adapt to hypoxia (29). In addition, studies have shown that Hsp70 can protect against tissue hypoxia and organ damage by degrading HIF-1 α activity under hypoxia (30), which leads to the increase of HSP70 level with altitude. To sum up, we can find that dairy cows will show varying degrees of adaptive physiological response with the increase of altitude.

In this study, we also predicted the involved pathways affected by altitude differences by using KEGG analyses. The differential metabolites involved in aminoacyl-tRNA biosynthesis were L-arginine, L-tryptophan, L-lysine, L-methionine, and L-proline. In this study, we found that the level of L-arginine increased with altitudes, and its rule of change is the same as that of NO (31), which might resulted from NO that could be synthesized from L-arginine (32). During hypoxia exposure, L-tryptophan levels in tissues increased significantly, which may be caused by blocked energy metabolism (33). Previous studies have shown that hypoxia can lead to the increase of lysine and methionine concentration (34), and it is consistent with the results of this experiment that lysine and methionine concentration increases at high altitude and very high altitude, which may be due to a disturbance of osmotic balance associated with hypoxia (35). A recent metabolic study has shown that a variety of amino acids, including proline, may be involved in the regulation of intracellular osmotic pressure during environmental hypoxia and may act as osmotic fluid (36), which might indicate that proline adapts to hypoxia as an osmolyte. These results suggested that dairy cows adapted to different altitudes by regulating the metabolic pathway of aminoacyl-tRNA biosynthesis.

In the present work, the pathway of valine, leucine, and isoleucine biosynthesis was both up-regulated in the CJ and XJ groups. Studies found that hypoxia contributed to the accumulation of L-valine (37), which was consistent with the results in the CJ group. However, there was lower abundance of L-valine in the XJ group, which might resulted from the application of valine to avoid mitochondrial damage or convert to other amino acids (38, 39). The differentially expressed metabolites of phenylalanine, tyrosine, and tryptophan biosynthetic signaling pathways in the CJ and XJ groups were tyrosine and phenylalanine, respectively. In the CJ group, the level of tyrosine increased, which might be due to the replenishment of insufficient energy supply to adapt to hypoxia stress (33). However, in the XJ group, the level of phenylalanine was reduced, which may be due to the more severe immune and inflammatory responses in cows at extremely high altitude (15), and phenylalanine needs to be converted into tyrosine to regulate oxidative stress, immune response, and inflammation, thus protecting the body from damage (40). These results suggest that dairy cows adapt to high-altitude hypoxia by upregulating phenylalanine metabolism and phenylalanine, tyrosine, and tryptophan biosynthetic signaling pathways.

Phytosphingosine and sphingosine are two important metabolites involved in sphingolipid metabolism. The results of this study showed that sphingolipid metabolism pathway was up-regulated under hypoxia. In the presence of hypoxia, elevated levels of plant sphingosine in the blood of cows in the CJ group (41) were found in this experiment, which might be caused by changes in key enzymes regulating sphingosine metabolism (42). The decreased level of sphingosine may be due to the conversion of blood sphingosine into plant sphingosine (43), which regulates angiogenesis in response to hypoxia stress (44). Additionally, we found that the sphingolipid metabolism pathway was up-regulated in dairy cows at high altitude compared with those at extremely high altitude, suggesting that sphingolipid metabolism might be negatively correlated with the adaptability to elevated altitude (42).

## Conclusion

In this experiment, we detected the related indexes of high-altitude adaptation in Jersey dairy cows in the GJ, CJ, and XJ groups, which replenished the basic data of blood biochemical indexes of Jersey dairy cows from different altitudes. At the same time, it was found that Jersey cows can adapt to high-altitude hypoxia mainly through up-regulation of amino acid metabolism and sphingolipid metabolism. Additionally, it was found that the metabolism of sphingolipid was negatively correlated with the ability to adapt to hypoxia induced by elevated altitudes.

## Data Availability Statement

The original contributions presented in the study are included in the article/Supplementary Material, further inquiries can be directed to the corresponding author/s.

## Ethics Statement

The animal study was reviewed and approved by the animal protection and use guidelines of Animal Protection Committee, Institute of Subtropical Agriculture, Chinese Academy of Sciences.

## Author Contributions

CZ, ZK, QH, and ZT made significant contributions in the conceptualization of the study. ZK, BL, and YZ made significant contributions in the analysis of data. ZK made significant contributions in the data curation and original draft preparation. CZ and ZT made significant contributions in the review and editing of the manuscript. CZ and BL made significant contributions in the funding acquisition. All authors have read and approved the final manuscript.

## Conflict of Interest

The authors declare that the research was conducted in the absence of any commercial or financial relationships that could be construed as a potential conflict of interest.

## Publisher's Note

All claims expressed in this article are solely those of the authors and do not necessarily represent those of their affiliated organizations, or those of the publisher, the editors and the reviewers. Any product that may be evaluated in this article, or claim that may be made by its manufacturer, is not guaranteed or endorsed by the publisher.
