# Supplementary material for: Comparative Analysis of Metabolic Differences of Jersey Cattle in Different High-Altitude Areas
Source: Front Vet Sci. 2021 Aug 3;8:713913. doi: 10.3389/fvets.2021.713913 (PMC8370252; doi:10.3389/fvets.2021.713913)
Supplement: Supplementary file 1 [file Data_Sheet_1.docx]

**Table S1-1 Summary of differentially expressedmetabolitesof CJ compared with GJ**

| **MS2 name** | **rt** | **mz** | **VIP** | **P-VALUE** | **FOLD CHANGE** |
| --- | --- | --- | --- | --- | --- |
| Choline | 30.5437 | 104.1074 | 1.5294 | 0.0180 | 2.6238 |
| 17-phenyl trinor Prostaglandin E2 serinol amide | 390.015 | 460.2693 | 1.4859 | 0.0242 | 5.3343 |
| tetranor-PGEM-d6 | 241.398 | 335.1997 | 1.3699 | 0.0008 | 1.8174 |
| Tryptophan | 201.763 | 205.0971 | 1.5732 | 0.0006 | 1.5375 |
| Arginine | 29.7890 | 175.1190 | 1.8007 | 0.0000 | 2.8363 |
| Phe Trp Ser Thr | 240.884 | 540.2411 | 1.1719 | 0.0004 | 2.6676 |
| 2-Hydroxycinnamic acid | 95.3457 | 165.0546 | 1.3827 | 0.0065 | 1.4117 |
| Valine | 52.4709 | 118.0865 | 1.6853 | 0.0001 | 2.0993 |
| Thiomorpholine 3-carboxylate | 42.2040 | 148.0426 | 1.0769 | 0.0482 | 1.2443 |
| (+)-4,11-Eudesmadien-3-one | 329.876 | 219.1743 | 1.1948 | 0.0181 | 1.8846 |
| Tyrosine | 95.2671 | 182.0812 | 1.3827 | 0.0056 | 1.4700 |
| Trimethylamine N-oxide | 32.2074 | 76.0764 | 1.7395 | 0.0039 | 3.9059 |
| Echinopsine | 167.249 | 160.0757 | 1.7621 | 0.0099 | 35.8361 |
| 6-Hydroxy-5-methoxyindole glucuronide | 213.715 | 340.1025 | 1.6758 | 0.0000 | 9.5599 |
| Isobutyryl carnitine | 192.110 | 232.1543 | 1.1334 | 0.0386 | 1.3777 |
| 3-ketosphinganine | 409.805 | 300.2896 | 1.7071 | 0.0087 | 3.8938 |
| 2-Methylbutyroylcarnitine | 219.334 | 246.1699 | 1.3695 | 0.0059 | 1.5527 |
| Nitroethane | 240.660 | 76.0400 | 1.6777 | 0.0001 | 1.9971 |
| Methioninesulfoxide | 60.5805 | 166.0533 | 1.5521 | 0.0011 | 1.4442 |
| 4-Oxoproline | 30.8645 | 130.0500 | 1.5233 | 0.0012 | 1.4105 |
| Clofop | 91.4441 | 293.0543 | 1.6767 | 0.0000 | 2.8907 |
| 7-(1,3-Cyclohexadienyl)-5-hydroxy-2,6-dimethyl-2-hepten-4-one | 455.530 | 235.1692 | 1.7689 | 0.0010 | 1.9292 |
| gamma-Glutamylleucine | 204.707 | 261.1444 | 1.4850 | 0.0024 | 2.0057 |
| Alpha-Pyrrolidinopropiophenone | 453.658 | 204.1383 | 1.7015 | 0.0002 | 2.4824 |
| PC(22:4(7Z,10Z,13Z,16Z)/0:0) | 463.870 | 572.3712 | 1.5545 | 0.0016 | 1.8458 |
| 3-Buten-1-amine | 35.401 | 72.0815 | 1.7292 | 0.0000 | 2.1768 |
| L-gamma-glutamyl-L-valine | 172.268 | 247.1287 | 1.6922 | 0.0001 | 2.1243 |
| Methionine | 61.629 | 150.0584 | 1.6063 | 0.0003 | 2.1971 |
| Calystegine A7 | 202.024 | 160.0950 | 1.6353 | 0.0001 | 1.8139 |
| PC(18:0/0:0) | 485.135 | 524.3713 | 1.3653 | 0.0116 | 1.4699 |
| Asperagenin | 444.835 | 449.3262 | 1.8452 | 0.0000 | 14.0348 |
| 3-methyl sulfolene | 91.431 | 133.0334 | 1.7084 | 0.0000 | 3.4334 |
| Serotonin | 167.279 | 177.1023 | 1.7569 | 0.0087 | 28.5427 |
| PC(22:6(4E,7E,10E,13E,16E,19E)/0:0)[U] | 447.826 | 568.3377 | 1.2458 | 0.0216 | 1.5146 |
| 1-(8Z,11Z,14Z-eicosatrienoyl)-sn-glycero-3-phosphocholine | 447.777 | 546.3557 | 1.3776 | 0.0085 | 1.6414 |
| Phenylacetylglycine | 240.670 | 194.0812 | 1.6685 | 0.0000 | 2.4810 |
| phenylacetylglutamine | 226.226 | 265.1182 | 1.7279 | 0.0000 | 8.5753 |
| Taccagenin | 486.129 | 447.3104 | 1.7867 | 0.0005 | 2.5434 |
| PC(7:0/O-8:0) | 419.297 | 482.3242 | 1.3510 | 0.0212 | 1.5865 |
| PC(0:0/20:4(5Z,8Z,11Z,14Z)) | 432.179 | 544.3400 | 1.4763 | 0.0046 | 1.6603 |
| Scalarin | 479.880 | 445.2948 | 1.7967 | 0.0006 | 95.1116 |
| C16 Sphingosine | 370.144 | 272.2582 | 1.7344 | 0.0061 | 4.0821 |
| PC(18:3(6Z,9Z,12Z)/0:0)[U] | 411.136 | 518.3244 | 1.5173 | 0.0047 | 1.7784 |
| Phytosphingosine | 364.543 | 318.3002 | 1.3112 | 0.0424 | 2.0365 |
| Lysine | 26.965 | 147.1128 | 1.7777 | 0.0000 | 2.7309 |
| PC(16:0/0:0)[U] | 438.421 | 496.3400 | 1.2893 | 0.0261 | 1.4450 |
| 2'-Aminoacetophenone | 95.476 | 136.0757 | 1.3190 | 0.0134 | 1.3804 |
| PC(15:1(9Z)/0:0) | 409.535 | 480.3086 | 1.3332 | 0.0111 | 2.1707 |
| Indole | 202.026 | 118.0654 | 1.3805 | 0.0098 | 1.2985 |
| 4-Deoxytetronic acid | 92.612 | 87.0447 | 1.7391 | 0.0011 | 2.1295 |
| gamma-Glutamyltyrosine | 182.892 | 311.1236 | 1.5850 | 0.0075 | 2.1541 |
| 2-hydroxy-butanoic acid | 92.531 | 105.0550 | 1.7172 | 0.0000 | 2.0972 |
| Ginkgotoxin | 249.140 | 184.0969 | 1.8320 | 0.0027 | 32.2052 |
| L-Arginine | 29.002 | 197.1010 | 1.7372 | 0.0000 | 2.1509 |
| 1,25-dihydroxy-10,19-methano-23-oxavitamin D3 | 482.779 | 433.3312 | 1.8575 | 0.0002 | 73.1638 |
| 1,25-Dihydroxy-24-oxo-16-ene-vitamin D3 | 485.535 | 429.2999 | 1.8052 | 0.0001 | 6.8670 |
| cis-4-Hydroxy-D-proline | 87.828 | 132.0658 | 1.5924 | 0.0007 | 0.5492 |
| piperidine | 34.342 | 86.0970 | 1.7648 | 0.0008 | 0.4720 |
| 4-Aminomethylcyclohexanecarboxylic acid | 34.1260 | 158.1176 | 1.7611 | 0.0038 | 0.0993 |
| Pipecolic acid | 34.061 | 130.0864 | 1.6695 | 0.0159 | 0.1354 |
| 3-(N-Nitrosomethylamino)propionitrile | 33.517 | 114.0665 | 1.6644 | 0.0002 | 0.6233 |
| DL-2-Aminooctanoic acid | 229.663 | 160.1332 | 1.4147 | 0.0251 | 0.4783 |
| Sphinganine | 398.038 | 302.3052 | 1.3867 | 0.0119 | 0.5381 |
| Vigabatrin | 55.184 | 130.0864 | 1.4384 | 0.0346 | 0.4920 |
| 2,8-Dihydroxyquinoline-beta-D-glucuronide | 216.697 | 338.0869 | 1.7712 | 0.0022 | 0.0913 |
| Difenoconazole | 440.554 | 406.0720 | 1.4183 | 0.0049 | 0.8597 |
| Phthalic anhydride | 437.880 | 149.0234 | 1.4427 | 0.0037 | 0.9246 |
| 1-Lauroyl-2-hydroxy-sn-glycero-3-phosphocholine | 367.017 | 440.2770 | 1.8140 | 0.0000 | 0.0026 |
| 5-Hydroxy-6-methoxyindole glucuronide | 228.260 | 340.1026 | 1.6578 | 0.0181 | 0.0611 |
| Bufadienolide | 372.469 | 355.2630 | 1.6919 | 0.0090 | 0.0942 |
| (3a,5b,7a)-23-carboxy-7-hydroxy-24-norcholan-3-yl, b-D-Glucopyranosiduronic acid | 370.224 | 569.3322 | 1.5900 | 0.0172 | 0.0347 |
| 2,5-Furandicarboxylic acid | 24.990 | 157.0150 | 1.1816 | 0.0215 | 0.7116 |
| Linoleamide | 504.506 | 280.2634 | 1.4341 | 0.0026 | 0.7240 |
| D-(+)-Turanose | 31.628 | 365.1053 | 1.6044 | 0.0034 | 0.0125 |
| Chol-11-Enic Acid | 372.573 | 373.2736 | 1.6881 | 0.0088 | 0.0975 |
| Citric acid | 69.384 | 193.0344 | 1.2712 | 0.0266 | 0.5789 |
| Asn Glu Leu Leu | 374.004 | 488.2679 | 1.7591 | 0.0008 | 0.3477 |
| Disulfiram | 450.470 | 297.0582 | 1.1108 | 0.0473 | 0.8786 |
| Trimethylaminoacetone | 233.185 | 116.1073 | 1.0957 | 0.0429 | 0.6701 |
| 5-Chola-3,11-dien-24-oic Acid | 369.410 | 357.2787 | 1.5926 | 0.0020 | 0.2594 |
| 2'-Deoxycytidine | 72.298 | 228.0978 | 1.4487 | 0.0118 | 0.6254 |
| Sphingofungin A | 381.490 | 432.3108 | 1.3975 | 0.0267 | 0.4051 |
| Cytidine | 61.883 | 244.0928 | 1.2202 | 0.0206 | 0.5559 |
| Homostachydrine | 207.278 | 158.1176 | 1.2209 | 0.0267 | 0.6384 |
| 5-Aminopentanamide | 216.140 | 117.1025 | 1.1408 | 0.0337 | 0.6459 |
| 13S-HpOTrE | 390.010 | 311.2216 | 1.8534 | 0.0001 | 0.0680 |
| Betonicine | 183.216 | 160.0968 | 1.7557 | 0.0025 | 0.2516 |
| Buprenorphine | 405.256 | 468.3085 | 1.6057 | 0.0003 | 0.5606 |
| Simvastatin acid | 475.055 | 437.2898 | 1.8609 | 0.0002 | 0.0304 |
| L-2-Amino-3-methylenehexanoic acid | 55.576 | 144.1019 | 1.8078 | 0.0057 | 0.0447 |
| 4-Carboxyphenylglycine | 253.282 | 196.0605 | 1.7575 | 0.0003 | 0.2055 |
| N4-Acetylcytidine | 181.540 | 286.1033 | 1.4770 | 0.0326 | 0.3699 |
| BENZALKONIUM | 422.911 | 304.2998 | 1.8293 | 0.0134 | 0.0257 |
| 7-Ketodeoxycholic acid | 500.635 | 424.3632 | 1.2251 | 0.0285 | 0.8610 |
| N-Nitroso-N-methylurethane | 53.517 | 133.0609 | 1.1027 | 0.0293 | 0.5750 |
| BISABOLOL ACETATE | 389.747 | 265.2162 | 1.8581 | 0.0001 | 0.0455 |
| trans-Aconitate | 69.384 | 175.0238 | 1.2104 | 0.0362 | 0.5620 |
| Methyl aminolevulinate | 80.505 | 146.0812 | 1.5406 | 0.0392 | 0.1174 |
| LysoPE(20:5(5Z,8Z,11Z,14Z,17Z)/0:0) | 428.524 | 500.2750 | 1.2425 | 0.0144 | 0.6754 |
| N,N-dimethyl-Safingol | 432.803 | 330.3365 | 1.4409 | 0.0215 | 0.4974 |
| N-Methylethanolamine phosphate | 30.860 | 156.0421 | 1.1125 | 0.0495 | 0.6615 |
| Se-Methylselenomethionine | 330.380 | 212.0198 | 1.6334 | 0.0270 | 0.0941 |
| Cholic Acid | 372.469 | 426.3213 | 1.7029 | 0.0054 | 0.1151 |
| N-arachidonoyl alanine | 372.372 | 376.2828 | 1.4807 | 0.0114 | 0.0558 |
| Carboxynorspermidine | 30.862 | 176.1394 | 1.4262 | 0.0016 | 0.5557 |

**TableS1-2Summary of differentially expressed metabolites inXJ compared with GJ**

| **MS2 name** | **rt** | **mz** | **VIP** | **P-VALUE** | **FOLD CHANGE** |
| --- | --- | --- | --- | --- | --- |
| Choline | 30.543 | 104.1074 | 1.3999 | 0.0111 | 2.3180 |
| Acetone oxime | 91.734 | 74.0608 | 1.6923 | 0.0000 | 9.5813 |
| tetranor-PGEM-d6 | 241.398 | 335.1997 | 1.2018 | 0.0049 | 1.6962 |
| Sphinganine | 398.038 | 302.3052 | 1.4282 | 0.0008 | 1.7611 |
| Arginine | 29.789 | 175.1190 | 1.5491 | 0.0001 | 2.2398 |
| 2-Acetylpyrazine | 27.840 | 123.0612 | 1.1761 | 0.0424 | 1.4375 |
| Valine | 52.470 | 118.0865 | 1.1395 | 0.0213 | 1.3945 |
| 6-Hydroxy-5-methoxyindole glucuronide | 213.715 | 340.1025 | 1.5081 | 0.0009 | 9.9496 |
| Urate-3-ribonucleoside | 177.639 | 301.0778 | 1.3208 | 0.0056 | 4.5951 |
| Nitroethane | 240.660 | 76.0400 | 1.0828 | 0.0196 | 1.2628 |
| 4-Oxoproline | 30.864 | 130.0500 | 1.2055 | 0.0102 | 1.2700 |
| DL-Stearoylcarnitine | 479.233 | 428.3734 | 1.4858 | 0.0003 | 2.8419 |
| gamma-Glutamylleucine | 204.707 | 261.1444 | 1.0947 | 0.0198 | 1.5240 |
| 6,9,12-Eicosatriynoic acid | 404.188 | 301.2161 | 1.5937 | 0.0005 | 5.9080 |
| 3-Buten-1-amine | 35.400 | 72.0815 | 1.2698 | 0.0043 | 1.4579 |
| Palmitoyl-L-carnitine | 448.579 | 400.3420 | 1.4919 | 0.0028 | 2.4235 |
| L-gamma-glutamyl-L-valine | 172.268 | 247.1287 | 1.3496 | 0.0024 | 1.6053 |
| O-ACETYL-L-CARNITINE | 34.073 | 204.1231 | 1.1379 | 0.0192 | 1.4795 |
| Linoelaidic Acid | 460.663 | 281.2474 | 1.3775 | 0.0167 | 3.5118 |
| Asperagenin | 444.835 | 449.3262 | 1.5337 | 0.0047 | 4.6455 |
| Acetylcarnitine | 65.936 | 204.1231 | 1.1856 | 0.0148 | 1.5032 |
| Phenylacetylglycine | 240.670 | 194.0812 | 1.0585 | 0.0220 | 1.3958 |
| phenylacetylglutamine | 226.226 | 265.1182 | 1.2924 | 0.0044 | 3.3176 |
| PC(0:0/20:4(5Z,8Z,11Z,14Z)) | 432.179 | 544.3400 | 1.0128 | 0.0438 | 1.3065 |
| Scalarin | 479.880 | 445.2948 | 1.6228 | 0.0090 | 76.5067 |
| PC(18:3(6Z,9Z,12Z)/0:0)[U] | 411.136 | 518.3244 | 1.0508 | 0.0391 | 1.3266 |
| Lysine | 26.965 | 147.1128 | 1.4544 | 0.0003 | 1.8684 |
| 9,12-octadecadienal | 481.097 | 265.2525 | 1.2036 | 0.0236 | 1.9838 |
| Petroselinic acid | 481.251 | 283.2630 | 1.1898 | 0.0242 | 1.7526 |
| gamma-Glutamyltyrosine | 182.892 | 311.1236 | 1.1835 | 0.0096 | 1.5645 |
| N,N-dimethyl-Safingol | 432.803 | 330.3365 | 1.5922 | 0.0000 | 3.3225 |
| Se-Methylselenomethionine | 330.380 | 212.0198 | 1.6910 | 0.0001 | 127.2338 |
| Elaidic carnitine | 456.356 | 426.3576 | 1.5940 | 0.0000 | 2.7530 |
| 1,25-dihydroxy-10,19-methano-23-oxavitamin D3 | 482.779 | 433.3312 | 1.2700 | 0.0343 | 15.1170 |
| cis-4-Hydroxy-D-proline | 87.828 | 132.0658 | 1.4301 | 0.0001 | 0.3483 |
| Trifluoperazine sulfoxide | 33.773 | 424.1672 | 1.7180 | 0.0000 | 0.0001 |
| Proline | 34.073 | 116.0709 | 1.0817 | 0.0198 | 0.7967 |
| piperidine | 34.342 | 86.0970 | 1.6740 | 0.0001 | 0.1221 |
| 17-phenyl trinor Prostaglandin E2 serinol amide | 390.015 | 460.2693 | 1.5545 | 0.0245 | 0.1875 |
| 5-AMINOPENTANOATE | 32.015 | 118.0865 | 1.1116 | 0.0168 | 0.8115 |
| 4-Aminomethylcyclohexanecarboxylic acid | 34.126 | 158.1176 | 1.5801 | 0.0047 | 0.1464 |
| Pipecolic acid | 34.061 | 130.0864 | 1.5144 | 0.0168 | 0.1475 |
| 3-(N-Nitrosomethylamino)propionitrile | 33.517 | 114.0665 | 1.4335 | 0.0005 | 0.6125 |
| Indoline | 175.538 | 120.0810 | 1.1078 | 0.0200 | 0.8012 |
| N,N-dimethyl-L-Valine | 192.917 | 146.1176 | 1.1137 | 0.0333 | 0.3621 |
| 3-Indoleacetic acid | 285.450 | 176.0706 | 1.6250 | 0.0028 | 0.1297 |
| Phenylalanine | 175.539 | 166.0863 | 1.1146 | 0.0189 | 0.8009 |
| Valdecoxib | 34.321 | 315.0797 | 1.6395 | 0.0002 | 0.0007 |
| Vigabatrin | 55.184 | 130.0864 | 1.5062 | 0.0173 | 0.3802 |
| Thelephoric acid | 36.205 | 353.0263 | 1.6259 | 0.0037 | 0.0026 |
| 3-Indolepropionic acid | 309.250 | 190.0862 | 1.4830 | 0.0482 | 0.0930 |
| Thiomorpholine 3-carboxylate | 42.204 | 148.0426 | 1.5785 | 0.0005 | 0.5267 |
| 1-Lauroyl-2-hydroxy-sn-glycero-3-phosphocholine | 367.017 | 440.2770 | 1.6111 | 0.0000 | 0.0397 |
| 5-Hydroxy-6-methoxyindole glucuronide | 228.260 | 340.1026 | 1.2893 | 0.0283 | 0.1717 |
| (+)-4,11-Eudesmadien-3-one | 329.876 | 219.1743 | 1.5824 | 0.0112 | 0.0900 |
| (3a,5b,7a)-23-carboxy-7-hydroxy-24-norcholan-3-yl, b-D-Glucopyranosiduronic acid | 370.224 | 569.3322 | 1.5161 | 0.0187 | 0.0574 |
| 2,5-Furandicarboxylic acid | 24.990 | 157.0150 | 1.6764 | 0.0001 | 0.1612 |
| Sphingosine-1-phosphate | 401.775 | 380.2559 | 1.1377 | 0.0222 | 0.8177 |
| (S,S)-Butane-2,3-diol | 3.744 | 73.0655 | 1.4823 | 0.0017 | 0.0866 |
| 2-Iodophenol methyl ether | 25.191 | 234.9611 | 1.7022 | 0.0005 | 0.0060 |
| 3-Guanidinopropanoate | 70.507 | 132.0769 | 1.5375 | 0.0078 | 0.3083 |
| 2-Pyrrolidinone | 72.327 | 86.0607 | 1.0434 | 0.0458 | 0.8153 |
| PE(18:2(9Z,12Z)/0:0) | 428.030 | 478.2929 | 1.5620 | 0.0000 | 0.4244 |
| Alpha-Pyrrolidinopropiophenone | 453.658 | 204.1383 | 1.2892 | 0.0060 | 0.6186 |
| Citric acid | 69.384 | 193.0344 | 1.4763 | 0.0031 | 0.3081 |
| PC(20:5(5Z,8Z,11Z,14Z,17Z)/0:0) | 432.000 | 542.3220 | 1.4049 | 0.0019 | 0.5993 |
| 1-heptadecanoyl-sn-glycero-3-phosphocholine | 463.041 | 510.3557 | 1.0855 | 0.0318 | 0.7247 |
| Acutifolane A | 383.963 | 263.1641 | 1.6446 | 0.0005 | 0.2597 |
| PC(17:1(9Z)/0:0) | 437.568 | 508.3400 | 1.0487 | 0.0406 | 0.7734 |
| D-Pantothenic acid | 186.448 | 220.1179 | 1.1979 | 0.0085 | 0.7100 |
| 2'-Deoxycytidine | 72.298 | 228.0978 | 1.4732 | 0.0035 | 0.5139 |
| Cytidine | 61.883 | 244.0928 | 1.4140 | 0.0061 | 0.4332 |
| LysoPE(15:0/0:0) | 416.226 | 440.2772 | 1.4596 | 0.0003 | 0.4587 |
| Prolyl-Hydroxyproline | 53.621 | 229.1183 | 1.6613 | 0.0001 | 0.3111 |
| Homostachydrine | 207.278 | 158.1176 | 1.4562 | 0.0056 | 0.4365 |
| 5-Aminopentanamide | 216.140 | 117.1025 | 1.7044 | 0.0006 | 0.0427 |
| 13S-HpOTrE | 390.010 | 311.2216 | 1.6959 | 0.0001 | 0.0295 |
| Betonicine | 183.216 | 160.0968 | 1.2459 | 0.0097 | 0.5089 |
| Buprenorphine | 405.256 | 468.3085 | 1.3898 | 0.0015 | 0.6158 |
| N-Methyl-2-oxoglutaramate | 33.799 | 160.0604 | 1.4110 | 0.0017 | 0.3967 |
| Simvastatin acid | 475.055 | 437.2898 | 1.6874 | 0.0002 | 0.0176 |
| L-2-Amino-3-methylenehexanoic acid | 55.576 | 144.1019 | 1.5510 | 0.0068 | 0.0948 |
| 4-Carboxyphenylglycine | 253.282 | 196.0605 | 1.4660 | 0.0000 | 0.1355 |
| N-Cinnamoylglycine | 273.930 | 206.0812 | 1.5242 | 0.0056 | 0.3279 |
| N4-Acetylcytidine | 181.540 | 286.1033 | 1.3327 | 0.0356 | 0.3863 |
| Taccagenin | 486.129 | 447.3104 | 1.1463 | 0.0219 | 0.4783 |
| BENZALKONIUM | 422.911 | 304.2998 | 1.6639 | 0.0136 | 0.0293 |
| BISABOLOL ACETATE | 389.747 | 265.2162 | 1.6992 | 0.0001 | 0.0330 |
| Proline-hydroxyproline | 34.074 | 229.1183 | 1.5990 | 0.0003 | 0.3438 |
| Asn Asp Gly Thr | 33.528 | 406.1567 | 1.6745 | 0.0020 | 0.0006 |
| trans-Aconitate | 69.384 | 175.0238 | 1.4937 | 0.0056 | 0.2967 |
| 2-O-[2-O-(alpha-D-Mannopyranosyl)-alpha-D-glucopyranosyl]-3-phospho-D-glycerate | 36.209 | 511.1085 | 1.6400 | 0.0001 | 0.0068 |
| PE(18:1(9Z)/0:0) | 454.460 | 480.3086 | 1.1946 | 0.0081 | 0.6941 |
| Methyl aminolevulinate | 80.505 | 146.0812 | 1.3737 | 0.0421 | 0.1366 |
| Lactamide | 30.811 | 90.0555 | 1.4419 | 0.0005 | 0.6727 |
| PE(16:0/0:0) | 442.975 | 454.2928 | 1.2972 | 0.0105 | 0.6565 |
| PC(O-16:0/3:1(2E)) | 482.059 | 536.3714 | 1.1610 | 0.0129 | 0.6460 |
| 2-Methylpropanal oxime | 158.686 | 88.0763 | 1.3590 | 0.0107 | 0.7401 |
| Ser Glu | 39.713 | 235.0924 | 1.6758 | 0.0042 | 0.0013 |
| LysoPE(20:5(5Z,8Z,11Z,14Z,17Z)/0:0) | 428.524 | 500.2750 | 1.5602 | 0.0000 | 0.3516 |
| 3-Hydroxy-1-indanone | 175.539 | 149.0598 | 1.0637 | 0.0264 | 0.8153 |
| N-Methylethanolamine phosphate | 30.860 | 156.0421 | 1.6057 | 0.0020 | 0.3096 |
| PC(P-17:0/0:0) | 478.380 | 494.3606 | 1.2370 | 0.0064 | 0.7839 |

**Table S1-3Summary of differentially expressed metabolites inCJ compared with XJ**

| **MS2 name** | **rt** | **mz** | **VIP** | **P-VALUE** | **FOLD CHANGE** |
| --- | --- | --- | --- | --- | --- |
| Trifluoperazine sulfoxide | 33.773 | 424.1672 | 1.6349 | 0.0000 | 13735.362 |
| Proline | 34.073 | 116.0709 | 1.1753 | 0.0080 | 1.2348 |
| piperidine | 34.342 | 86.0970 | 1.5542 | 0.0000 | 3.8642 |
| 17-phenyl trinor Prostaglandin E2 serinol amide | 390.015 | 460.2693 | 1.5608 | 0.0131 | 28.4509 |
| Met His Lys | 405.256 | 415.2114 | 1.2714 | 0.0171 | 1.9604 |
| Creatine | 34.066 | 132.0768 | 1.1939 | 0.0071 | 1.1314 |
| Indoline | 175.538 | 120.0810 | 1.3710 | 0.0006 | 1.3370 |
| Tryptophan | 201.763 | 205.0971 | 1.2364 | 0.0072 | 1.3042 |
| 3-Indoleacetic acid | 285.450 | 176.0706 | 1.5749 | 0.0013 | 6.2184 |
| Phenylalanine | 175.539 | 166.0863 | 1.3532 | 0.0008 | 1.3407 |
| Valdecoxib | 34.321 | 315.0797 | 1.5636 | 0.0000 | 1325.799 |
| Vigabatrin | 55.184 | 130.0864 | 1.1465 | 0.0285 | 1.2941 |
| Arginine | 29.789 | 175.1190 | 1.0325 | 0.0191 | 1.2663 |
| Thelephoric acid | 36.205 | 353.0263 | 1.5620 | 0.0001 | 367.3213 |
| Phe Trp Ser Thr | 240.884 | 540.2411 | 1.2245 | 0.0068 | 1.1559 |
| 2-Hydroxycinnamic acid | 95.345 | 165.0546 | 1.4849 | 0.0011 | 1.6394 |
| 3-Indolepropionic acid | 309.250 | 190.0862 | 1.3438 | 0.0085 | 4.1400 |
| Valine | 52.470 | 118.0865 | 1.3466 | 0.0013 | 1.5054 |
| Thiomorpholine 3-carboxylate | 42.204 | 148.0426 | 1.5609 | 0.0002 | 2.3626 |
| (+)-4,11-Eudesmadien-3-one | 329.876 | 219.1743 | 1.5902 | 0.0003 | 20.9292 |
| Tyrosine | 95.267 | 182.0812 | 1.5141 | 0.0003 | 1.7095 |
| 2,5-Furandicarboxylic acid | 24.990 | 157.0150 | 1.5533 | 0.0005 | 4.4137 |
| (S,S)-Butane-2,3-diol | 3.744 | 73.0655 | 1.3947 | 0.0001 | 8.4337 |
| Echinopsine | 167.249 | 160.0757 | 1.4186 | 0.0119 | 13.0002 |
| 2-Iodophenol methyl ether | 25.191 | 234.9611 | 1.6215 | 0.0005 | 138.0844 |
| 3-ketosphinganine | 409.805 | 300.2896 | 1.5180 | 0.0069 | 4.7121 |
| Nitroethane | 240.660 | 76.0400 | 1.4765 | 0.0019 | 1.5815 |
| Methioninesulfoxide | 60.580 | 166.0533 | 1.5025 | 0.0000 | 1.6451 |
| 3-Guanidinopropanoate | 70.507 | 132.0769 | 1.4533 | 0.0043 | 2.6618 |
| Clofop | 91.444 | 293.0543 | 1.1315 | 0.0072 | 1.7300 |
| 7-(1,3-Cyclohexadienyl)-5-hydroxy-2,6-dimethyl-2-hepten-4-one | 455.530 | 235.1692 | 1.5265 | 0.0007 | 1.9258 |
| PE(18:2(9Z,12Z)/0:0) | 428.030 | 478.2929 | 1.1414 | 0.0234 | 1.8139 |
| Alpha-Pyrrolidinopropiophenone | 453.658 | 204.1383 | 1.5531 | 0.0004 | 4.0133 |
| Citric acid | 69.384 | 193.0344 | 1.2666 | 0.0011 | 1.8788 |
| Melleolide M | 405.258 | 453.1674 | 1.2931 | 0.0122 | 2.0016 |
| PE(18:0/0:0) | 486.914 | 482.3242 | 1.1644 | 0.0150 | 1.6810 |
| PC(22:4(7Z,10Z,13Z,16Z)/0:0) | 463.870 | 572.3712 | 1.1110 | 0.0239 | 1.4475 |
| dodecanamide | 433.028 | 200.2009 | 1.0653 | 0.0366 | 1.3784 |
| PC(20:5(5Z,8Z,11Z,14Z,17Z)/0:0) | 432.000 | 542.3220 | 1.2852 | 0.0044 | 1.4050 |
| 1-heptadecanoyl-sn-glycero-3-phosphocholine | 463.041 | 510.3557 | 1.1780 | 0.0140 | 1.6414 |
| Acutifolane A | 383.963 | 263.1641 | 1.5966 | 0.0000 | 3.7332 |
| 3-Buten-1-amine | 35.400 | 72.0815 | 1.3591 | 0.0012 | 1.4931 |
| L-gamma-glutamyl-L-valine | 172.268 | 247.1287 | 1.0842 | 0.0223 | 1.3232 |
| PC(17:1(9Z)/0:0) | 437.568 | 508.3400 | 1.1515 | 0.0093 | 1.4085 |
| Methionine | 61.629 | 150.0584 | 1.5442 | 0.0003 | 3.1485 |
| 7C-aglycone | 280.930 | 299.1277 | 1.0453 | 0.0111 | 2.1381 |
| Calystegine A7 | 202.024 | 160.0950 | 1.2720 | 0.0028 | 1.3961 |
| 2'-Deoxycytidine | 72.298 | 228.0978 | 1.0554 | 0.0271 | 1.2169 |
| LysoPE(15:0/0:0) | 416.226 | 440.2772 | 1.3257 | 0.0016 | 2.2211 |
| Prolyl-Hydroxyproline | 53.621 | 229.1183 | 1.5978 | 0.0000 | 3.3940 |
| Homostachydrine | 207.278 | 158.1176 | 1.0620 | 0.0154 | 1.4627 |
| 5-Aminopentanamide | 216.140 | 117.1025 | 1.6239 | 0.0003 | 15.1205 |
| PC(18:0/0:0) | 485.135 | 524.3713 | 1.2745 | 0.0048 | 1.6596 |
| 13S-HpOTrE | 390.010 | 311.2216 | 1.4054 | 0.0002 | 2.3048 |
| Asperagenin | 444.835 | 449.3262 | 1.4556 | 0.0000 | 3.0211 |
| 3-methyl sulfolene | 91.431 | 133.0334 | 1.0904 | 0.0101 | 1.8341 |
| Serotonin | 167.279 | 177.1023 | 1.4151 | 0.0103 | 12.4543 |
| N-Methyl-2-oxoglutaramate | 33.799 | 160.0604 | 1.3246 | 0.0008 | 2.1886 |
| Simvastatin acid | 475.055 | 437.2898 | 1.0492 | 0.0395 | 1.7334 |
| LysoPC(18:2(9Z,12Z)) | 427.910 | 520.3400 | 1.2524 | 0.0298 | 1.5971 |
| PC(22:6(4E,7E,10E,13E,16E,19E)/0:0)[U] | 447.826 | 568.3377 | 1.1806 | 0.0115 | 1.5667 |
| N-Cinnamoylglycine | 273.930 | 206.0812 | 1.2662 | 0.0410 | 2.3565 |
| 1-(8Z,11Z,14Z-eicosatrienoyl)-sn-glycero-3-phosphocholine | 447.777 | 546.3557 | 1.2904 | 0.0052 | 1.6940 |
| Phenylacetylglycine | 240.670 | 194.0812 | 1.5013 | 0.0001 | 1.7774 |
| phenylacetylglutamine | 226.226 | 265.1182 | 1.3535 | 0.0002 | 2.5848 |
| Taccagenin | 486.129 | 447.3104 | 1.3959 | 0.0000 | 5.3180 |
| PC(7:0/O-8:0) | 419.297 | 482.3242 | 1.2896 | 0.0100 | 1.7864 |
| Proline-hydroxyproline | 34.074 | 229.1183 | 1.5391 | 0.0000 | 2.7936 |
| C16 Sphingosine | 370.144 | 272.2582 | 1.5261 | 0.0057 | 4.2482 |
| Asn Asp Gly Thr | 33.528 | 406.1567 | 1.5894 | 0.0007 | 970.4232 |
| Phytosphingosine | 364.543 | 318.3002 | 1.2049 | 0.0359 | 2.1468 |
| Lysine | 26.965 | 147.1128 | 1.3119 | 0.0012 | 1.4616 |
| trans-Aconitate | 69.384 | 175.0238 | 1.3093 | 0.0017 | 1.8939 |
| 4'-Apo-beta,psi-caroten-4'-al | 462.265 | 483.3645 | 1.3044 | 0.0046 | 1.4081 |
| 2-O-[2-O-(alpha-D-Mannopyranosyl)-alpha-D-glucopyranosyl]-3-phospho-D-glycerate | 36.209 | 511.1085 | 1.5121 | 0.0107 | 140.6532 |
| PC(16:0/0:0)[U] | 438.421 | 496.3400 | 1.2190 | 0.0245 | 1.4949 |
| 2'-Aminoacetophenone | 95.476 | 136.0757 | 1.5050 | 0.0013 | 1.6035 |
| Indole | 202.026 | 118.0654 | 1.1413 | 0.0199 | 1.1980 |
| PE(18:1(9Z)/0:0) | 454.460 | 480.3086 | 1.0017 | 0.0379 | 1.3939 |
| 4-Deoxytetronic acid | 92.612 | 87.0447 | 1.0929 | 0.0132 | 1.5923 |
| Lactamide | 30.811 | 90.0555 | 1.4442 | 0.0001 | 1.5872 |
| PE(16:0/0:0) | 442.975 | 454.2928 | 1.1777 | 0.0204 | 1.6550 |
| PC(O-16:0/3:1(2E)) | 482.059 | 536.3714 | 1.3290 | 0.0009 | 1.8360 |
| 2-Methylpropanal oxime | 158.686 | 88.0763 | 1.3271 | 0.0348 | 1.9594 |
| Ser Glu | 39.713 | 235.0924 | 1.6004 | 0.0009 | 1124.6477 |
| LysoPE(20:5(5Z,8Z,11Z,14Z,17Z)/0:0) | 428.524 | 500.2750 | 1.2409 | 0.0050 | 1.9209 |
| 2-hydroxy-butanoic acid | 92.531 | 105.0550 | 1.2542 | 0.0019 | 1.7167 |
| 3-Hydroxy-1-indanone | 175.539 | 149.0598 | 1.3405 | 0.0011 | 1.3196 |
| N-Methylethanolamine phosphate | 30.860 | 156.0421 | 1.2921 | 0.0100 | 2.1365 |
| L-Arginine | 29.002 | 197.1010 | 1.0112 | 0.0019 | 1.9071 |
| 1,25-dihydroxy-10,19-methano-23-oxavitamin D3 | 482.779 | 433.3312 | 1.2022 | 0.0001 | 4.8398 |
| 1,25-Dihydroxy-24-oxo-16-ene-vitamin D3 | 485.535 | 429.2999 | 1.4311 | 0.0000 | 7.5280 |
| Acetone oxime | 91.734 | 74.0608 | 1.6147 | 0.0000 | 0.1406 |
| 4-(Trimethylammonio)but-2-enoate | 34.045 | 144.1019 | 1.3229 | 0.0088 | 0.4780 |
| O-Acetylethanolamine | 33.828 | 104.0710 | 1.1596 | 0.0097 | 0.6217 |
| Sphinganine | 398.038 | 302.3052 | 1.5114 | 0.0000 | 0.3056 |
| Palmitic amide | 522.632 | 256.2634 | 1.0052 | 0.0268 | 0.7212 |
| 2-Acetylpyrazine | 27.840 | 123.0612 | 1.0578 | 0.0312 | 0.6925 |
| Docosahexaenoic Acid ethyl ester | 426.620 | 357.2786 | 1.3820 | 0.0332 | 0.1397 |
| Difenoconazole | 440.554 | 406.0720 | 1.0284 | 0.0229 | 0.8755 |
| Phthalic anhydride | 437.880 | 149.0234 | 1.1404 | 0.0094 | 0.9299 |
| 5-Hydroxy-6-methoxyindole glucuronide | 228.260 | 340.1026 | 1.3994 | 0.0076 | 0.3558 |
| Bufadienolide | 372.469 | 355.2630 | 1.3457 | 0.0386 | 0.1607 |
| Linoleamide | 504.506 | 280.2634 | 1.1528 | 0.0114 | 0.6667 |
| D-(+)-Turanose | 31.628 | 365.1053 | 1.4356 | 0.0164 | 0.0057 |
| 3-keto Petromyzonol | 426.921 | 375.2893 | 1.3495 | 0.0427 | 0.1123 |
| Chol-11-Enic Acid | 372.573 | 373.2736 | 1.3438 | 0.0368 | 0.1656 |
| DL-Stearoylcarnitine | 479.233 | 428.3734 | 1.2694 | 0.0041 | 0.5359 |
| 6,9,12-Eicosatriynoic acid | 404.188 | 301.2161 | 1.6049 | 0.0005 | 0.0873 |
| Asn Glu Leu Leu | 374.004 | 488.2679 | 1.2101 | 0.0310 | 0.3190 |
| LysoPC(20:1(11Z)) | 505.592 | 550.3870 | 1.1241 | 0.0297 | 0.6030 |
| Palmitoyl-L-carnitine | 448.579 | 400.3420 | 1.1195 | 0.0358 | 0.5248 |
| Linoelaidic Acid | 460.663 | 281.2474 | 1.3387 | 0.0184 | 0.2920 |
| Betonicine | 183.216 | 160.0968 | 1.3607 | 0.0177 | 0.4944 |
| 3-Oxo-5-chola-7,9(11)-dien-24-oic Acid | 349.893 | 371.2579 | 1.5101 | 0.0114 | 0.0173 |
| 9,12-octadecadienal | 481.097 | 265.2525 | 1.1569 | 0.0271 | 0.5177 |
| Petroselinic acid | 481.251 | 283.2630 | 1.0238 | 0.0397 | 0.6246 |
| 3-Hydroxy-12-oxo-5-chol-6-en-24-oic Acid | 349.892 | 389.2685 | 1.5002 | 0.0098 | 0.0238 |
| N,N-dimethyl-Safingol | 432.803 | 330.3365 | 1.6133 | 0.0000 | 0.1497 |
| Se-Methylselenomethionine | 330.380 | 212.0198 | 1.6174 | 0.0001 | 0.0007 |
| Cholic Acid | 372.469 | 426.3213 | 1.3574 | 0.0250 | 0.1834 |
| N-arachidonoyl alanine | 372.372 | 376.2828 | 1.1716 | 0.0449 | 0.1030 |
| Elaidic carnitine | 456.356 | 426.3576 | 1.3634 | 0.0031 | 0.3910 |
| Carboxynorspermidine | 30.862 | 176.1394 | 1.1474 | 0.0101 | 0.5108 |
