# Supplementary material for: Comparative Analysis of Metabolic Differences of Jersey Cattle in Different High-Altitude Areas
Source: Front Vet Sci. 2021 Aug 3;8:713913. doi: 10.3389/fvets.2021.713913 (PMC8370252; doi:10.3389/fvets.2021.713913)
Supplement: Supplementary file 2 [file Data_Sheet_2.docx]

**Table S2-1 Summary of differentially expressed pathways of CJ compared with GJ**

| **Pathway** | **Hits** | **-ln(p)** | **FDR** | **Impact** |
| --- | --- | --- | --- | --- |
| Aminoacyl-tRNA biosynthesis | 6 | 5.0386 | 0.5251 | 0 |
| Sphingolipid metabolism | 3 | 4.0277 | 0.7215 | 0.22557 |
| Ubiquinone and other terpenoid-quinone biosynthesis | 1 | 2.5283 | 1 | 0 |
| Phenylalanine, tyrosine and tryptophan biosynthesis | 1 | 2.2539 | 1 | 0.5 |
| Biotin metabolism | 1 | 2.044 | 1 | 0 |
| Glycerophospholipid metabolism | 2 | 1.6797 | 1 | 0.07122 |
| Phenylalanine metabolism | 1 | 1.5086 | 1 | 0 |
| Valine, leucine and isoleucine biosynthesis | 1 | 1.3338 | 1 | 0.33333 |
| Pyrimidine metabolism | 2 | 1.3163 | 1 | 0.02167 |
| Tryptophan metabolism | 2 | 1.1731 | 1 | 0.32134 |
| Pantothenate and CoA biosynthesis | 1 | 1.0747 | 1 | 0 |
| Pentose and glucuronate interconversions | 1 | 1.0747 | 1 | 0.25 |
| Glyoxylate and dicarboxylate metabolism | 1 | 1.0228 | 1 | 0.2963 |
| Lysine degradation | 1 | 0.84966 | 1 | 0 |
| Propanoate metabolism | 1 | 0.84966 | 1 | 0 |
| Citrate cycle (TCA cycle) | 1 | 0.84966 | 1 | 0.05356 |
| Starch and sucrose metabolism | 1 | 0.74679 | 1 | 0.03789 |
| Cysteine and methionine metabolism | 1 | 0.61046 | 1 | 0.09464 |
| Glycine, serine and threonine metabolism | 1 | 0.52422 | 1 | 0 |
| Valine, leucine and isoleucine degradation | 1 | 0.42165 | 1 | 0 |
| Tyrosine metabolism | 1 | 0.36665 | 1 | 0.14548 |
| Arginine and proline metabolism | 1 | 0.34234 | 1 | 0.0765 |
| Primary bile acid biosynthesis | 1 | 0.31987 | 1 | 0 |

**Table S2-2 Summary of differentially expressed pathways of XJ compared with GJ**

| **Pathway** | **Hits** | **-ln(p)** | **FDR** | **Impact** |
| --- | --- | --- | --- | --- |
| Aminoacyl-tRNA biosynthesis | 5 | 3.8177 | 1 | 0 |
| Pantothenate and CoA biosynthesis | 2 | 2.8939 | 1 | 0.02041 |
| Phenylalanine, tyrosine and tryptophan biosynthesis | 1 | 2.3058 | 1 | 0.5 |
| Biotin metabolism | 1 | 2.0952 | 1 | 0 |
| Phenylalanine metabolism | 1 | 1.557 | 1 | 0.40741 |
| Pyrimidine metabolism | 2 | 1.3941 | 1 | 0.02167 |
| Valine, leucine and isoleucine biosynthesis | 1 | 1.3809 | 1 | 0.33333 |
| Arginine and proline metabolism | 2 | 1.1507 | 1 | 0.15438 |
| Pentose and glucuronate interconversions | 1 | 1.1192 | 1 | 0.25 |
| Glyoxylate and dicarboxylate metabolism | 1 | 1.0666 | 1 | 0.2963 |
| Lysine degradation | 1 | 0.89094 | 1 | 0 |
| Citrate cycle (TCA cycle) | 1 | 0.89094 | 1 | 0.05356 |
| Sphingolipid metabolism | 1 | 0.85389 | 1 | 0.14286 |
| Starch and sucrose metabolism | 1 | 0.78625 | 1 | 0.03789 |
| Glycerophospholipid metabolism | 1 | 0.62326 | 1 | 0.02442 |
| Glycine, serine and threonine metabolism | 1 | 0.55856 | 1 | 0 |
| Valine, leucine and isoleucine degradation | 1 | 0.45285 | 1 | 0 |
| Fatty acid metabolism | 1 | 0.43772 | 1 | 0 |
| Tryptophan metabolism | 1 | 0.40926 | 1 | 0 |

**Table S2-3 Summary of differentially expressed pathways of CJ compared with XJ**

| **Pathway** | **Hits** | **-ln(p)** | **FDR** | **Impact** |
| --- | --- | --- | --- | --- |
| Aminoacyl-tRNA biosynthesis | 8 | 8.0842 | 0.0249 | 0 |
| Phenylalanine, tyrosine and tryptophan biosynthesis | 2 | 5.3674 | 0.1889 | 1 |
| Sphingolipid metabolism | 3 | 3.8891 | 0.5174 | 0.22557 |
| Phenylalanine metabolism | 2 | 3.667 | 0.5174 | 0.40741 |
| Ubiquinone and other terpenoid-quinone biosynthesis | 1 | 2.4785 | 1 | 0 |
| Tryptophan metabolism | 3 | 2.2014 | 1 | 0.32134 |
| Arginine and proline metabolism | 3 | 2.0426 | 1 | 0.16547 |
| Biotin metabolism | 1 | 1.9956 | 1 | 0 |
| Valine, leucine and isoleucine biosynthesis | 1 | 1.2895 | 1 | 0.33333 |
| Pantothenate and CoA biosynthesis | 1 | 1.0331 | 1 | 0 |
| Pentose and glucuronate interconversions | 1 | 1.0331 | 1 | 0.25 |
| Glyoxylate and dicarboxylate metabolism | 1 | 0.9818 | 1 | 0.2963 |
| Propanoate metabolism | 1 | 0.81111 | 1 | 0 |
| Citrate cycle (TCA cycle) | 1 | 0.81111 | 1 | 0.05356 |
| Starch and sucrose metabolism | 1 | 0.71004 | 1 | 0.03789 |
| Cysteine and methionine metabolism | 1 | 0.57657 | 1 | 0.09464 |
| Glycerophospholipid metabolism | 1 | 0.55394 | 1 | 0.0468 |
| Glycine, serine and threonine metabolism | 1 | 0.49251 | 1 | 0 |
| Pyrimidine metabolism | 1 | 0.40777 | 1 | 0.01032 |
